# Morphology of brood pouch formation in the pot-bellied seahorse *Hippocampus abdominalis*

**DOI:** 10.1186/s40851-017-0080-9

**Published:** 2017-10-17

**Authors:** Mari Kawaguchi, Ryohei Okubo, Akari Harada, Kazuki Miyasaka, Kensuke Takada, Junya Hiroi, Shigeki Yasumasu

**Affiliations:** 1 Department of Materials and Life Sciences, Faculty of Science and Technology, Sophia University, 7–1 Kioi-cho, Chiyoda-ku, Tokyo, 102–8554 Japan; 2 Department of Anatomy, St. Marianna University School of Medicine, 2-16-1 Sugao, Miyamae-ku, Kawasaki, 216–8511 Japan

**Keywords:** Seahorse, Brood pouch, Morphology, Immunohistochemistry, Development

## Abstract

**Background:**

The reproductive strategies of vertebrates are diverse. Seahorses (Pisces: Syngnathidae) possess the unique characteristic of male pregnancy; i.e., males, not females, incubate embryos in a specialized structure called a ‘brood pouch’. The brood pouch is formed along the ventral midline of the tail. The lumen of the brood pouch is surrounded by loose connective tissue, called pseudoplacenta, and dermis.

**Results:**

We visualized and evaluated the morphology of brood pouch formation in *Hippocampus abdominalis* to gain generalizable insights into this process in seahorses. First, we employed several staining methods to characterize the pseudoplacenta and dermis of the brood pouch of mature male seahorses. The pseudoplacenta is composed mainly of reticular fibers, while the dermis is composed mainly of collagenous fibers. Further observations showed that pouch formation is initiated by linear projections of epithelia on both ventrolateral sides of the body. These projections elongated toward the ventral midline, eventually fused together, and then formed a baggy structure composed of a single dermis layer with neither smooth muscle nor pseudoplacenta. Finally, the pseudoplacenta was formed, together with two layers of dermis and smooth muscle. Thus, a fully developed brood pouch was established. The morphology of the luminal epithelium also changed during pouch formation. We analyzed the localization of C-type lectins as markers; haCTL II was localized in both the outer and luminal epithelia of the brood pouch throughout development in the male seahorse, whereas haCTL IV, which was not detected in the early stage of seahorse development, became localized only in the luminal epithelium as development proceeded.

**Conclusions:**

We categorized the processes of brood pouch formation during male seahorse development into three stages: (1) the early stage, characterized by formation of a baggy structure from the primordium; (2) the middle stage, characterized by the differentiation and establishment of brood pouch-specific tissues; and (3) the late stage, characterized by a fully formed pouch with developing blood vessels and a pouch fold ultimately capable of carrying and incubating embryos.

**Electronic supplementary material:**

The online version of this article (10.1186/s40851-017-0080-9) contains supplementary material, which is available to authorized users.

## Backround

Reproductive strategies vary greatly among vertebrate groups. Fishes in particular exhibit a wide variety of reproductive modes. For example, some species of gobies and sticklebacks protect their eggs until hatching by making nests, and some species of cichlids protect eggs and hatched-out fry within their mouths [1]. Species of Syngnathidae, the seahorses and pipefishes, share a particularly unique reproductive strategy [2], in which male possesses an embryo-incubating structure called a ‘brood pouch’, into which the female deposits eggs. The brood pouch is formed on either the abdomen (subfamily Gastrophori) or tail (subfamily Urophori) of the male [3]. The anatomy of this reproductive structure varies from species to species. A simple structure is that in which eggs are loosely attached without protective plates or membrane, while the complicated structure is a pocket-like structure in which eggs are incorporated [4, 5], and these unique organs are found only in the syngnathids. Following the terminology used in mammalians, this reproductive strategy in fishes is called ‘male pregnancy’ [6]. The brood pouch is not present in juvenile seahorses, and develops during post-juvenile growth.

The female seahorse deposits eggs into the brood pouch and the male ejaculates sperm at the entrance of the pouch. Fertilization occurs immediately during egg transfer [7]. The male retains the embryos until development is complete [8]. During incubation, embryos are enclosed in brood pouch tissue called pseudoplacenta [9], and the morphology of the pseudoplacenta is known to change after the transfer of eggs from female [10]. Embryos are finally embedded within individual compartments formed in pseudoplacenta [11], and there, the number of blood vessels increases. The brood pouch, like the mammalian placenta, performs the functions of gas exchange, waste removal, and osmoregulation [9, 12–14]. Carcupino et al. [13] examined brood pouch skin morphology in three species of syngnathids and found that, despite certain differences, the dermis was commonly vascularized. Previous studies have mainly described the precise morphology of the brood pouch before, during, or after the incubation of embryos. In the present study, we focused on the detailed successive, developmental processes of brood pouch formation of seahorses.

The pseudoplacenta is rich in blood vessels, suggesting father-to-embryo interaction through epithelia on the lumen, similar to mother-to-embryo interaction in mammalian placenta. The epithelium facing the lumen of the pouch is considered to be derived from epithelium that covers the outser surface of the body. To better understand the timing of such changes of the outer epithelium into luminal epithelium, we sought to identify molecular markers that are expressed specifically in the luminal epithelium. It has recently been reported that, in *H. comes*, C-type lectins are expressed in the brood pouch; however, their precise expression sites have not yet been determined. C-type lectins are in a type of carbohydrate-binding protein, the activity of which is mediated by conserved carbohydrate recognition domains (CRDs). Analyses of complimentary DNA (cDNA) libraries made from *Hippocampus kuda* and *H. comes* revealed that C-type lectins were commonly abundant in brood pouch of both species [15, 16]. The cloned C-type lectin composed of a single CRD belonged to group VII C-type lectins. In the present study, we found that one of the C-type lectins (haCTL IV) is specifically localized in the luminal epithelium, while another (haCTL II) is commonly localized in both the outer and luminal epithelia of the pouch. Thus, we analyzed these C-type lectins to monitor the development of brood pouch epithelium.

In the present study, we observed brood pouch formation in the pot-bellied seahorse *Hippocampus abdominalis*. To understand the pathway of pouch formation, we first characterized brood pouch tissues in mature male seahorses by employing hematoxylin and eosin staining and Masson’s trichrome staining to identify fibrous collagen. The latter was used to stain *H. kuda* brood pouch dermis [10]. We further employed reticulin silver staining to identify reticular fibers, Elastica van Gieson staining to identify elastic fibers, and immunohistochemical staining to identify C-type lectins. Based on our morphological observations, we propose plausible histological and gene-expression details in brood pouch formation during male post-juvenile seahorse development.

## Methods

### Fish

Pot-bellied seahorses, *Hippocampus abdominalis*, aged 20–30 days (20 individuals), two months (two individuals), three months (one individual), five months (one individual), six months (one individual), seven months (three individuals), and eight months (two individuals), as well as several mature adults, were obtained from Seahorse Ways Co. Ltd., Kagoshima, Japan, for use in the present study. Seahorses were anesthetized in ethyl 3-aminobenzoate methanesulfonate (MS-222) to obtain tissues. This study was performed in accordance with the guidelines of the Animal Experiment Committee of Sophia University, Tokyo.

### Histology

Body portions including the brood pouch were fixed in 4% paraformaldehyde in phosphate-buffered saline (PBS) at 4 °C for 24 h. The samples were dehydrated in a stepwise manner using 25, 50, 70, 90, 95 and 100% ethanol, and then embedded in paraffin following the standard protocol [17]. Thin (8-μm) sections were cut with a Microm HM 325 Rotary Microtome (Thermo Scientific™), deparaffinized in Clear-Rite 3 (Thermo Scientific™ Richard-Allan Scientific™), and stained using the standard protocols for hematoxylin and eosin stain, Masson’s trichrome stain, reticulin silver stain, and Elastica van Gieson stain [17]. After staining, the samples were observed under a light microscope (BX51, Olympus, Tokyo, Japan) equipped with a digital camera (D7000, Nikon, Tokyo, Japan).

For hematoxylin and eosin staining, thin sections were first stained in Mayer hematoxylin solution for 10 min and then rinsed in distilled water. Counterstaining was done in eosin solution for 10 min, followed by dehydrating the sections using 70% and 100% alcohol for 5 min each, then twice for 5 min each using 100% alcohol. After clearing in Clear-Rite 3, the sections were mounted with ClearVue™ Mountant XYL (Thermo Fisher Scientific).

For Masson’s trichrome staining, sections were deparaffinized and rehydrated through 100%, 95%, and 70% alcohol. After washing in distilled water, sections were stained in Weigert’s iron hematoxylin solution for 5 min. After washing in distilled water, sections were stained in Biebrich scarlet-acid fuchsin solution for 5 min. After washing in distilled water, sections were immersed in 2.5% phosphomolybdic-2.5% phosphotungstic acid solution for 30 min. The sections were then transferred to aniline blue solution and stained for 5 min, rinsed briefly in distilled water, and immersed in 1% acetic acid solution for 5 min. After washing in distilled water, the sections were dehydrated through 100% isopropyl alcohol, and finally cleared in Clear-Rite 3 prior to mounting.

Reticulin silver staining was performed with a Reticulum II Staining Kit (Roche) according to the manufacturer’s protocol. In brief, the deparaffinized and rehydrated sections were oxidized in acidified potassium permanganate solution for 3 min, rinsed in distilled water, decolorized with 1% oxalic acid for 2 min, and again rinsed in distilled water. Next, the sections were treated with 2% ammonium iron sulfate, rinsed in distilled water, and impregnated with silver carbonate solution. After rinsing again in distilled water, the sections were reduced with 0.4% formalin for 10 min, then treated with 1% gold chloride overnight. After fixing with 2% aqueous sodium thiosulfate for 5 min and washing in water for 5 min, the sections were counterstained with nuclear fast red for 1 min.

Elastica van Gieson staining was performed with an Elastic Stain Kit (Sigma-Aldrich, St. Louis, MO, USA) according to the manufacturer’s protocol. In brief, deparaffinized and rehydrated sections were treated with elastic stain solution for 10 min, differentiated in ferric chloride for 5 min, and treated with van Gieson’s counterstain.

### Cloning of C-type lectin cDNA

First-strand cDNA was synthesized from RNA extracted from the brood pouch of a mature male using a SMART™ RACE cDNA Amplification Kit (Clontech Laboratories, Mountain View, CA, USA). 5′ RACE PCR and its nested PCR, and 3′-RACE PCR were performed using gene-specific primers designed from nucleotide sequences of the three C-type lectin (CTL) fragments obtained from PCR using primers designed in conserved regions of *Hippocampus comes* CTL genes. Nucleotide sequences of the primers are as follows:

haCTL I 5′-RACE (for 1st PCR): 5′-ATCGGTCCATATGTAGTCTTCAGCCAGCAG-3′.

haCTL I 5′-RACE (for nested PCR): 5′-GTCAGGAGGAGGATCATTCTGGAAGGTGGT-3′.

haCTL I 3′-RACE: 5′-GGAGCCTGGTCTTTCAAAAAGATTCAGTCC-3′.

haCTL II 5′-RACE (for 1st PCR): 5′-GTGATGATGGAGCTTCATTGTGACTGAAGG-3′.

haCTL II 5′-RACE (for nested PCR): 5′-GCTCTTGGACGTTACAAATTGGTTGTCAGG-3′.

haCTLII 3′-RACE: 5′-AAATTCAACGACCTTCTCTGTCCTAAAGGG-3′.

haCTL IV 5′-RACE (for 1st PCR): 5′-GGTGTATTTCACAACTGAGCCATCGGTCCA-3′.

haCTL IV 5′-RACE (for nested PCR): 5′-CTGCCAAAACCCTTCGAGTCTCGAAAACTG-3′.

haCTL IV 3′-RACE: 5′-GGACAACCGCTGTTACATCTACCAGAGTCC-3′.

### Phylogenetic analysis

We searched for homologous genes for C-type lectin (haCTL I, II, and IV) with the BLAST program from GenBank using their amino acid sequences. In total, 89 genes were used in the phylogenetic analysis. The accession numbers are listed in Additional file 1: Figure S1. Amino acid sequences of the carbohydrate-recognition domain (CRD) were aligned using the ClustalX [18] program. Maximum-likelihood (ML) analysis, using a WAG model with I + Γ, was conducted with RAxML version 8.2.10 [19]. We reconstructed a ML tree, with simultaneous bootstrap replicates (1000), to determine the best-scoring topology.

### Semi-quantitative RT-PCR expression analysis

One mature male seahorse was dissected to isolate the liver, intestine, heart, gill, muscle, brain, eye, gallbladder, kidney, and brood pouch, and one mature female seahorse was dissected to isolate the ovaries. The RNAs were extracted using RNAiso (Takara Bio Inc., Otsu, Japan) according to the manufacturer’s instructions. The brood pouch sample isolated from a second mature seahorse was divided into two parts: the dermis along with outer epithelium, and the pseudoplacenta along with the luminal epithelium. Next, for cDNA construction, we used 0.5 μg of the RNAs to synthesize cDNA using PrimeScript Reverse Transcriptase (Takara Bio Inc.) and a poly-dT primer. The cDNA fragments were amplified with EmeraldAmp® PCR Master Mix (Takara Bio Inc.) using the primer sets shown below. The cDNA amounts added to the PCR cocktail were normalized by the intensity of the amplified β-actin band.

haCTL I-F: 5′-GGAGCCTGGTCTTTCAAAAAGATTCAGTCC-3′.

haCTL I-R: 5′-GTTGCAACAACAGTGTGGCAAGG-3′.

haCTL II-F: 5′-TCGTTCAGCAACTGATTGTCGCC-3′.

haCTL II-R: 5′-GACTGGAGAGATGCTCTTGGACG-3′.

haCTL IV-F: 5′-TCTGGTCTCCATTCGTGATACCG-3′.

haCTL IV-R: 5′-TTGCCAATAATTCAGCATCGTGG-3′.

β-actin-F 5′-ACACCTTCTACAACGAGCTG-3′.

β-actin-R 5′-GTACAGGTCCTTACGGATGT-3′.

### Antibody

The haCTL II and haCTL IV were composed of a signal peptide and the CRD. The CRD was amplified from haCTL IV cDNA, using primers containing the restriction enzyme sites BamHI and NdeI, and cloned into the pET3c vector for expression in *Escherichia coli* strain BL21 with a C-terminal His tag. Nucleotide sequences of the primers are as follows:

F: 5′-CATATGTGGAATCGGTGTCCACATGGCTGG-3′.

R: 5′-GGATCCTTAATGATGATGATGATGGATGCCAGAGCCGCCAAGGAATTT-3′.

Inclusion bodies (insoluble protein aggregates), obtained according to a previously described method [20], were dissolved into a denaturing buffer (50 mM Tris–HCl pH 8.0, 8 M urea, 0.1 M 2-mercaptoethanol, and 1 mM EDTA). The clear protein solution thus obtained was diluted 1000-fold with a refolding buffer (50 mM Tris–HCl pH 8.0, 0.8 M L-arginine hydrochloride, 1 mM glutathione, 0.1 mM oxidized glutathione, and 1 mM CaCl_2_) and incubated at 4 °C for two days. This protein solution was dialyzed four times against 25 mM Tris–HCl buffer (pH 8.0) with 1 mM CaCl_2_. The folding mixture was loaded onto an Ni-NTA Superflow (QIAGEN Inc., Valencia, CA, USA) and eluted once with 20 mM Tris–HCl buffer (pH 8) containing 0.3 M imidazole and 0.15 M NaCl.

Inclusion bodies of haCTL II were not obtained according to the above method. Instead, the CRD of haCTL II was amplified by PCR using primers containing restriction enzyme sites (EcoRI at the 5′ end, and Sall at the 3′ end), and then ligated into the pMAL-c2X vector (New England Biolabs, Beverly, MA, USA) to obtain a fusion protein of haCTL II and maltose-binding protein (MBP). Nucleotide sequences of the primers are as follows:

F: 5′-GAATTCCCTTCAAAAAAATTAAACGACCTT-3′.

R: 5′-GTCGACTTAGTGGAACAAACGGTGCGCTTC-3′.

*E. coli* strain BL21 containing expression plasmids were cultured, and then cell lysate was collected following a previously described method [20]. Because the haCTL II and MBP fusion protein was obtained as water-soluble protein, the supernatant of the cell lysate was applied to amylose resin and eluted once with 20 mM Tris–HCl buffer (pH 7.4) containing 0.2 M NaCl and 1 mM EDTA. The eluted protein was further purified with Source 15Q column (GE Healthcare Bio-Sciences Corp., Piscataway, NJ, USA) using a high-performance liquid chromatography system.

The recombinant proteins were dialyzed against PBS, mixed with an equivalent volume of Complete Freund’s Adjuvant, and injected into a rabbit (for haCTL II) or a mouse (for haCTL IV) three times at 2-week intervals. One week after the last immunization, antisera were obtained. Specificity of the antisera against the haCTL II or haCTL IV was checked by western blotting using recombinant proteins.

### Immunohistochemistry

Sections of two late stage and one early stage animals were first placed sequentially in each of the following treatments: two times, 10 min each, Clear-Rite 3; two times, 5 min each, 100% ethanol; 5 min, 95% ethanol; 5 min, 70% ethanol. Sections were then washed in water for 5 min, and blocked in blocking buffer (1% bovine serum albumin in PBS) for 1 h. Incubation with a primary antibody (1/100 dilution in blocking buffer) was performed for 1 h, then sections were washed three times with PBS for 5 min each. Thereafter, sections were incubated for 30 min in 500-fold dilution of alkaline phosphatase (AP)-conjugated secondary antibody (Thermo Fisher Scientific) in blocking buffer, and then washed three times in PBS for 5 min each. Finally, AP protein was visualized by adding NBT/BCIP solution to the sections for 10 min, then washing for 5 min in PBS. Once stained, the samples were observed with a light microscope (BX51, Olympus) equipped with a digital camera (D7000, Nikon).

## Results

### Histology of the mature brood pouch

**Fig. 1:**
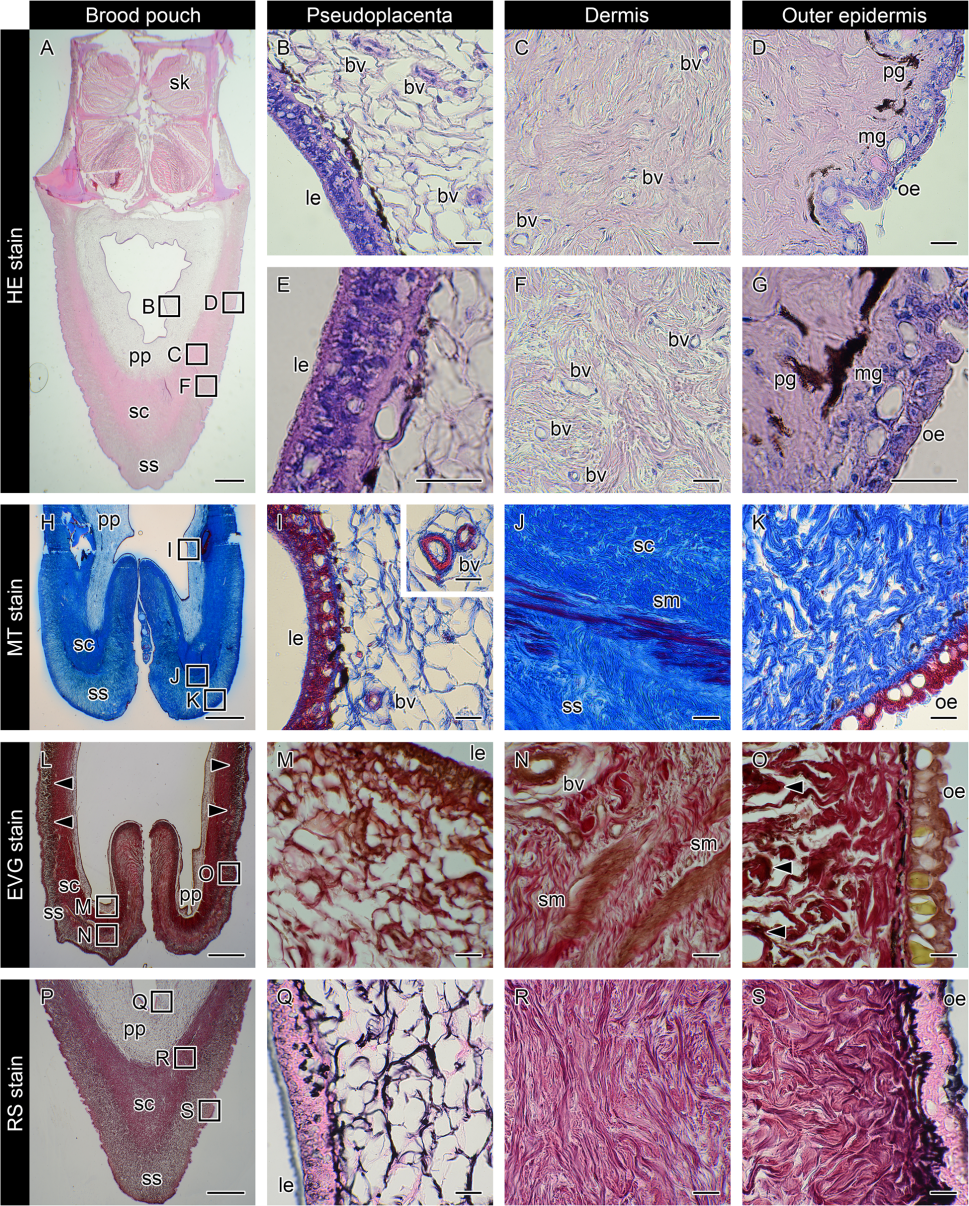
Histology of the brood pouch in mature pot-bellied seahorse *Hippocampus abdominalis*. Hematoxylin and eosin (HE) stain of cross-sections of the brood pouch (**a**–**g**), Masson’s trichrome (MT) stain of a cross-section around the entrance of the pouch (**h**–**k**), Elastica van Gieson (EVG) stain of a cross-section around the entrance of the pouch (**l**–**o**), and reticulin silver (RS) stain of a cross-section of the brood pouch (**p**–**s**). The dorsal side is at the top and the ventral side is at the bottom in **a**, **h**, **l**, and **p**. The lettered boxes in **a**, **h**, **l** and **p** indicate sites of high magnification of pseudoplacenta (**b**, **i**, **m**, **q**), dermis (**c**, **f**, **j**, **n**, **r**), and outer epidermis (**d**, **k**, **o**, **s**). High-magnification views of pseudoplacenta (**e**) and outer epidermis (**g**). The inset panel in (**i**) shows the large blood vessels found in pseudoplacenta. Arrowheads indicate black signals after EVG staining. Scale bars: **a**, **h**, **l**, ***p*** = 1 mm; **b**–**g**, **i**–**k**, **m**–**o**, and **q**–**s** = 20 μm. bv, blood vessel; le, luminal epithelium; mg, mucous granule; oe, outer epithelium; pg, pigment cell; pp, pseudoplacenta; sc, stratum compactum; sk, skeletal muscle; sm, smooth muscle; ss, stratum spongiosum. These photographs were taken using two individual male seahorses; one specimen for **a**, **h**, **p**, and another for **l**, due to the lack of black staining in the first specimen

The brood pouch of mature *Hippocampus abdominalis* males is located along the ventral midline of the tail posterior to the anus. We first observed the histology of the pouch after hematoxylin and eosin (HE) staining. As shown in the cross-section in Fig. 1a, the pouch is localized on the ventral skeletal-muscle masses of the tail. Both the skeletal muscle and the pouch were surrounded by outer epithelium. The outer epithelium was composed of stratified cuboidal epithelial cells with many large mucous granules (Fig. 1d, g). The lumen of the pouch was surrounded by three layers: two distinct dermis layers and the pseudoplacenta (Fig. 1a). Beneath the basement membrane of the epithelium, pigment cells were observed (Fig. 1g). The dermis of the brood pouch is composed of two layers: the outer layer or stratum spongiosum (Fig. 1a, f), and the inner layer or stratum compactum (Fig. 1a, c) [21]. The stratum spongiosum consists of a loose network of connective tissue, while the stratum compactum consists of tightly connected collagenous fibers. Numerous blood vessels were observed in both layers of the dermis (Fig. 1c, f). The pseudoplacenta is located beneath the stratum compactum (Fig. 1a) and composed of connective tissue with a mesh-patterned structure and abundant blood vessels (Fig. 1b). The lumen of the pouch is surrounded by luminal epithelium (Fig. 1b, e). No notable mucous granules were found in the luminal epithelium (Fig. 1b). We examined four mature males, and all males showed the morphology described above.

**Fig. 2:**
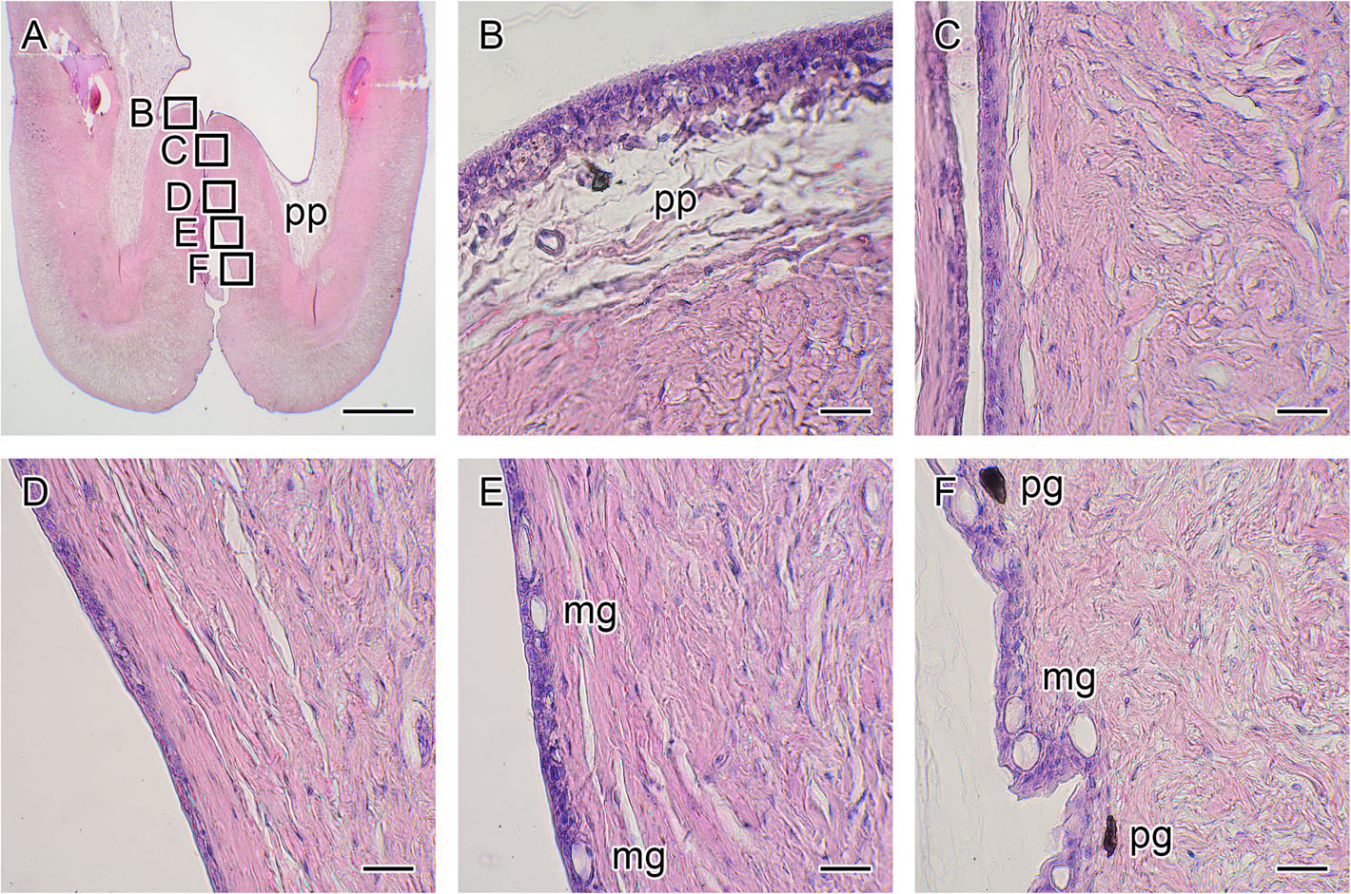
Hematoxylin and eosin staining around the entrance of the seahorse brood pouch. A cross-section of the brood pouch around the entrance is shown in **a**. The dorsal side is at the top and the ventral side is at the bottom. The lettered boxes in **a** indicate sites of high-magnification views of the luminal epithelium (**b**) to the outer epithelium (**c**–**f**). mg, mucous granule; pg, pigment cell; pp, pseudoplacenta. Scale bars: **a** = 1 mm; **b**–**f** = 20 μm. The same individual (also depicted in Fig. 1 **a**) is shown in all photographs

The lumen of the brood pouch opens to the outside through a small pore at the anterior of the pouch. We further observed the epithelial layer around the entrance of the pouch after HE staining (Fig. 2). No notable mucous granules were observed within the luminal epithelium (Fig. 2b); however, some mucous granules were observed in the epithelium around the middle of the entrance (Fig. 2e), and the number of granules was increased toward the outer epithelium (Fig. 2f).

### Characterization of the brood pouch dermis and pseudoplacenta by means of three staining methods

The dermis of the brood pouch in *H. kuda* has been stained blue using Masson’s trichrome (MT) [10]. MT also stained the two dermis layers in *H. abdominalis*: the outer stratum spongiosum was stained light blue (Fig. 1h, k), while the inner stratum compactum was stained dark blue due to its higher fiber content (Fig. 1h, j). The blue staining of the pseudoplacenta (Fig. 1h, i) was much weaker than that of the dermis layers. Blood vessels within the pseudoplacenta were clearly recognized with red staining (Fig. 1i). Smooth muscle was observed between the stratum compactum and stratum spongiosum, and it was especially abundant around the pouch entrance (Fig. 1j). We observed four mature males, all of which showed similar characteristics, corresponding well to observations reported in the previous investigations of *H. kuda* [10].

Next, we further employed Elastica van Gieson staining on samples of four adult seahorses (Fig. 1l). In all individuals, smooth muscle within the dermis was clearly recognized as brown staining (Fig. 1n). In two of four individuals, black stain, denoting elastic fibers, was observed in the outer stratum spongiosum (Fig. 1o), while the other two individuals displayed no black staining. These results suggest that a small amount of elastic fibers is present in the stratum spongiosum, with the composition varying among individuals.

Finally, reticulin silver staining clearly distinguished pseudoplacenta from dermis (Fig. 1p). The fibers of pseudoplacenta were stained black (Fig. 1q), signifying reticular fibers, whereas the two dermis layers (stratum compactum, Fig. 1r; stratum spongiosum, Fig. 1s) were mainly stained red-purple, signifying collagenous fibers. Some black staining was also observed near the epithelial basement membrane (Fig. 1q, s). These observations were consistent across all four mature males studied. These results suggest that the pseudoplacenta is composed mainly of reticular fibers, while the two dermis layers appear to comprise mainly collagenous fibers.

### Formation of the brood pouch

**Fig. 3:**
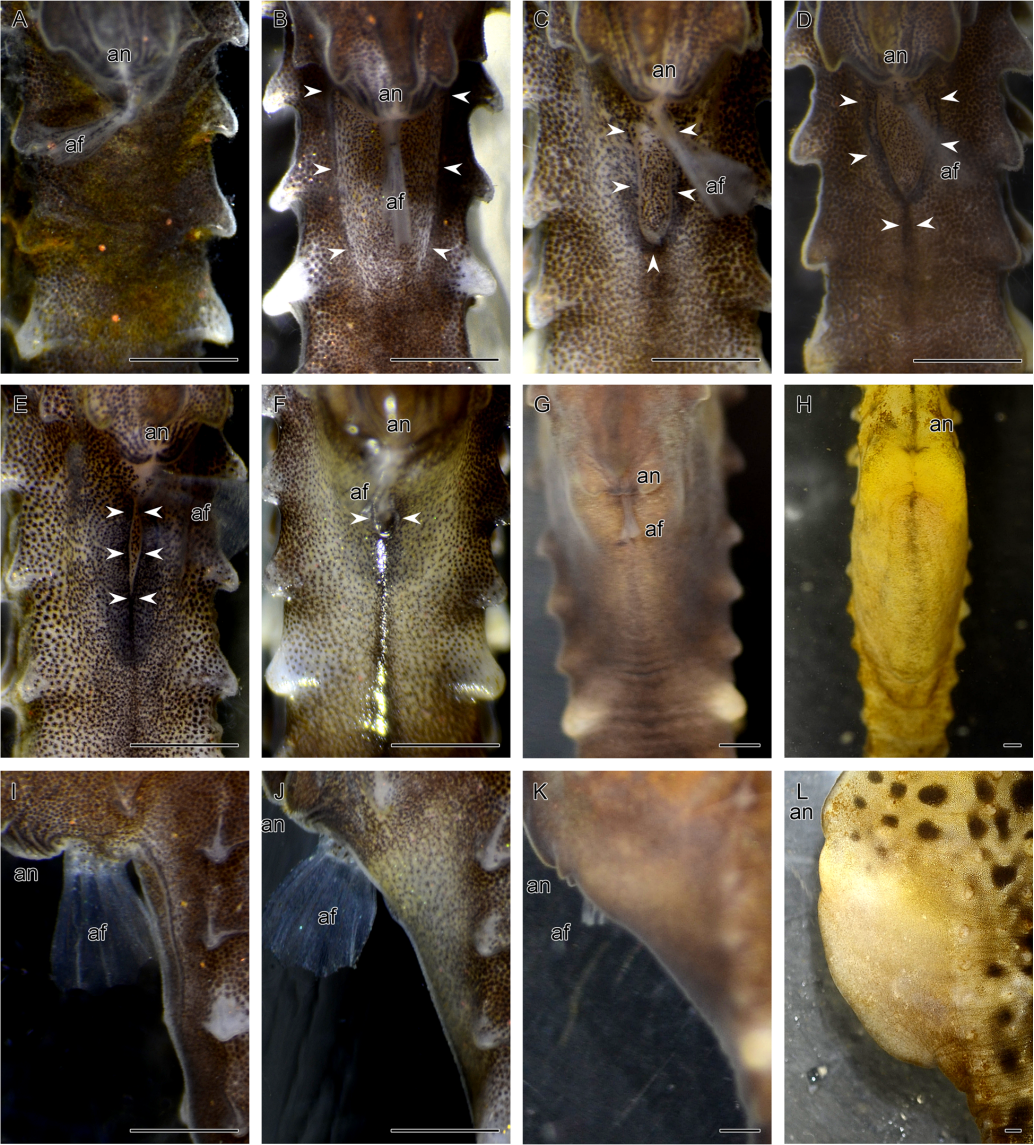
External observations during seahorse brood pouch formation. Ventral views (**a**–**h**) and lateral views (**i**–**l**) of the tail of a male seahorse, observed 20–30 days (**a**–**f**, **i**, **j**), five months (**g**, **k**), and eight months (**h**, **l**) after birth. (**a**) Before formation of the brood pouch primordia. The order of brood pouch development is depicted in **b**–**f**: (**b**, **i**) primordium of the brood pouch as parallel linear projections; (**c**) elongation of the projections; (**d**) a seam line forming a Y-shape; (**e**) a seam line forming an I-shape; (**f**, **j**) formation of the pore-like pouch entrance; the expanding pouch as development proceeded to five months (**g**, **k**) and eight months (**h**, **l**) after birth. Arrowheads point to edges of the brood pouch. af, anal fin; an, anus. Scale bars: 1 mm

To understand how the brood pouch is established during the development of the male seahorse, we observed 20 juveniles (body length 5.6 ± 0.7 cm) 20–30 days after birth. In 10 of 20 fish, structures regarded as part of the early stage of brood pouch formation were observed on the ventral side of the tail. The remaining 10 animals showed no visible signs of a brood pouch, thus making their sex identification impossible, and two of them began to form primordia of the pouch within one week of the initial observation. The brood pouch formation process was observed as follows: In the earliest stage, a white plate structure was observed just posterior to the anus (indicated with white arrowheads in Fig. 3b). This structure was surrounded with linear projections on both ventrolateral sides (Fig. 3i). As development proceeded, the projections seemed to elongate toward the ventral midline of the body, and the caudal part of the projection could be clearly observed (Fig. 3c). Both sides of the elongated projections fused together at the midline of the posterior end. Thus, a Y-shaped seam line was formed (Fig. 3d). As fusion proceeded, the connected part was observed as an I-shaped seam line (Fig. 3e). Finally, the baggy structure of the brood pouch was formed, and only a pore (the hole-shaped entrance) remained just posterior to the anus (Fig. 3f, j). As development proceeded, the baggy structure expanded (Fig. 3g, k: brood pouch in a 5-month-old individual) until the brood pouch appeared fully developed ready for incubation of embryos (Fig. 3h, l: brood pouch in an 8-month-old individual).

### Stages of brood pouch formation

Based on our observations of the various developmental stages of the brood pouch in males ranging 1–8 months in age, we classified the brood pouch formation into three progressive stages.

**Fig. 4:**
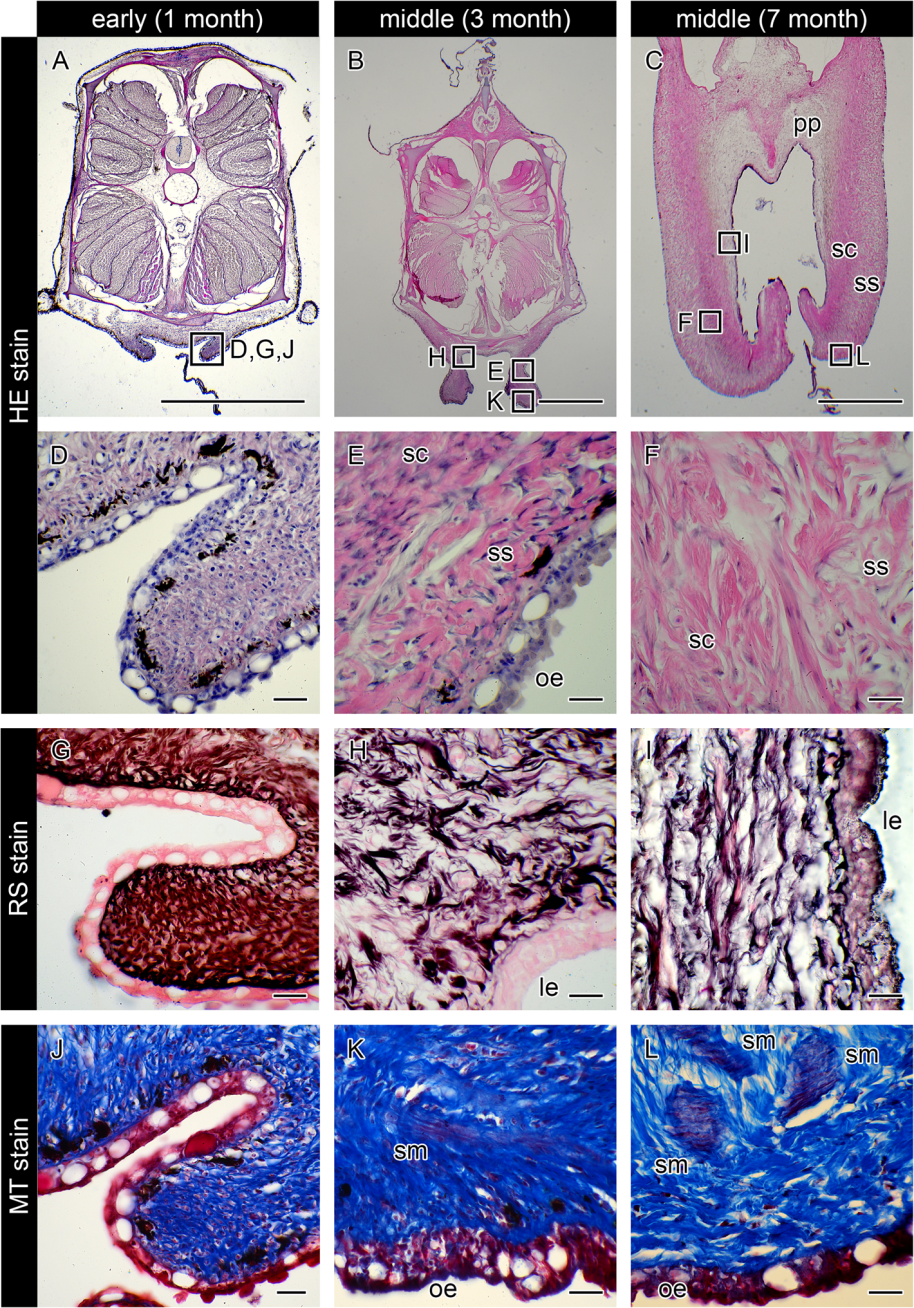
Histological observations of seahorse brood pouch formation. Cross-sections of the brood pouch were observed by various staining methods: hematoxylin and eosin (HE) stain (**a**–**f**), reticulin silver (RS) stain (**g**–**i**), and Masson’s trichrome (MT) stain (**j**–**l**). These photographs show three individual males: one specimen for **a**, **d**, **g**, **j**, at 20–30 days after birth, showing the Y-shaped seam line (the same individual as in Fig. 3 **d**); one specimen for **b**, **e**, **h**, **k**, at three months after birth; and one specimen for **c**, **f**, **i**, **l**, at seven months after birth. The lettered boxes in **a**–**c** indicate the corresponding sites of high-magnification views shown in **d**–**l**. Scale bars: **a**–**c** = 1 mm; **d**–**l** = 20 μm. le, luminal epithelium; oe, outer epithelium; pp, pseudoplacenta; sc, stratum compactum; sm, smooth muscle; ss, stratum spongiosum

**Fig. 5:**
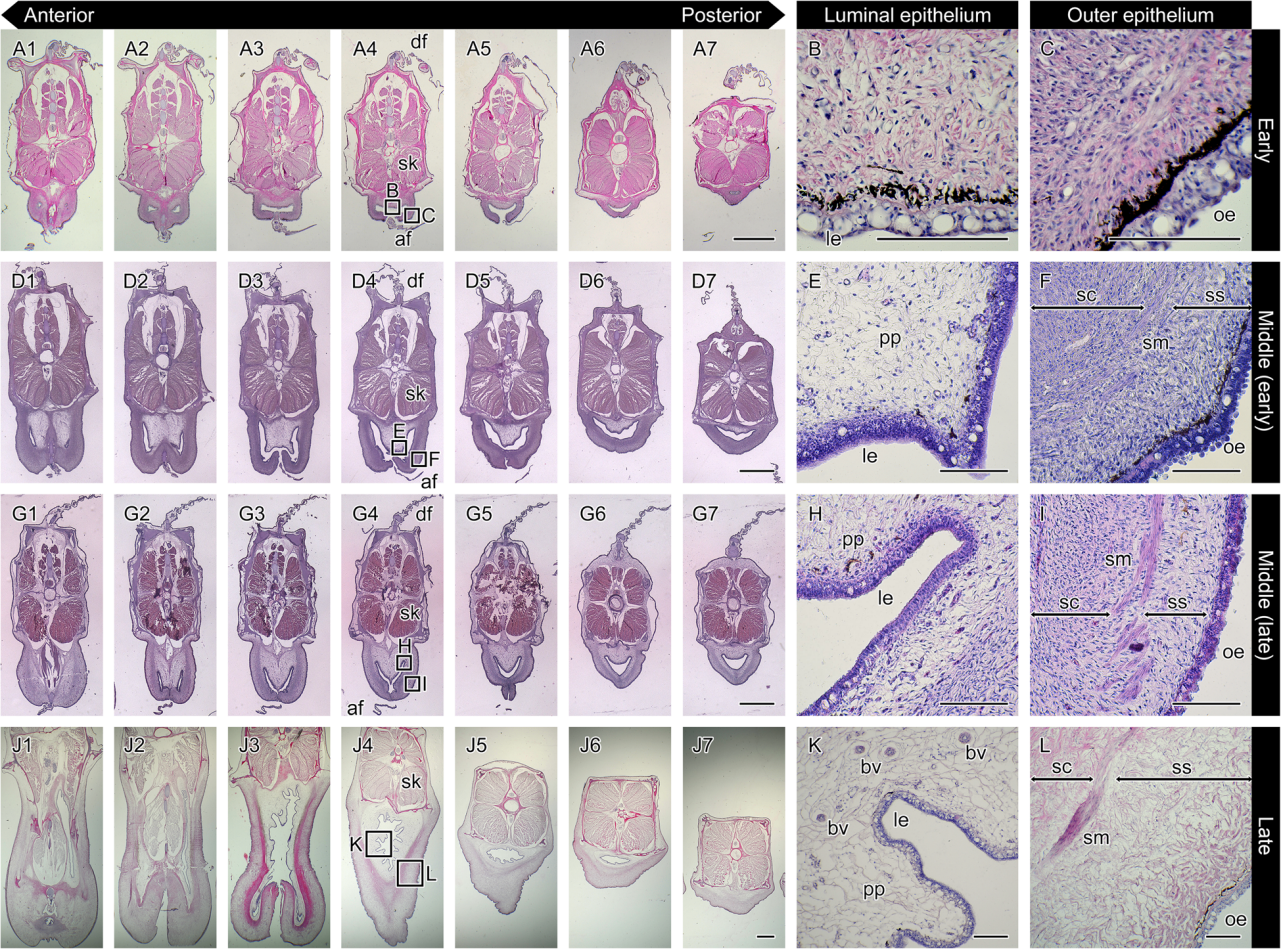
Hematoxylin and eosin stain of serial sections throughout development of the seahorse brood pouch. Serial sections of the body: at the early stage of pouch development showing the I-shaped seam line (**a**1–7; the same individual as in Fig. 3**e**); in the early period of the middle stage (**d**1–7), and the latter period of the middle stage (**g**1–7); and at the late stage (**j**1–7). The section views are from the anterior (1) to posterior (7). The lettered boxes in **a**4, **d**4, **g**4, and **j**4 indicate sites of high magnification of the luminal epithelium (**b**, **e**, **h**, **k**) and outer epithelium (**c**, **f**, **i**, **l**). af, anal fin; bv, blood vessel; df, dorsal fin; le, luminal epithelium; oe, outer epithelium; pp, pseudoplacenta; sc, stratum compactum; sk, skeletal muscle; sm, smooth muscle; ss, stratum spongiosum. Scale bars: **a**, **d**, **g**, **j** = 1 mm; **b**, **c**, **e**, **f**, **h**, **i**, **k**, **l** = 100 μm. These photographs were taken using four individuals: one specimen for **a**–**c**, one for **d**–**f**, one for **g**–**i**, and one for **j**–**l**

**Fig. 6:**
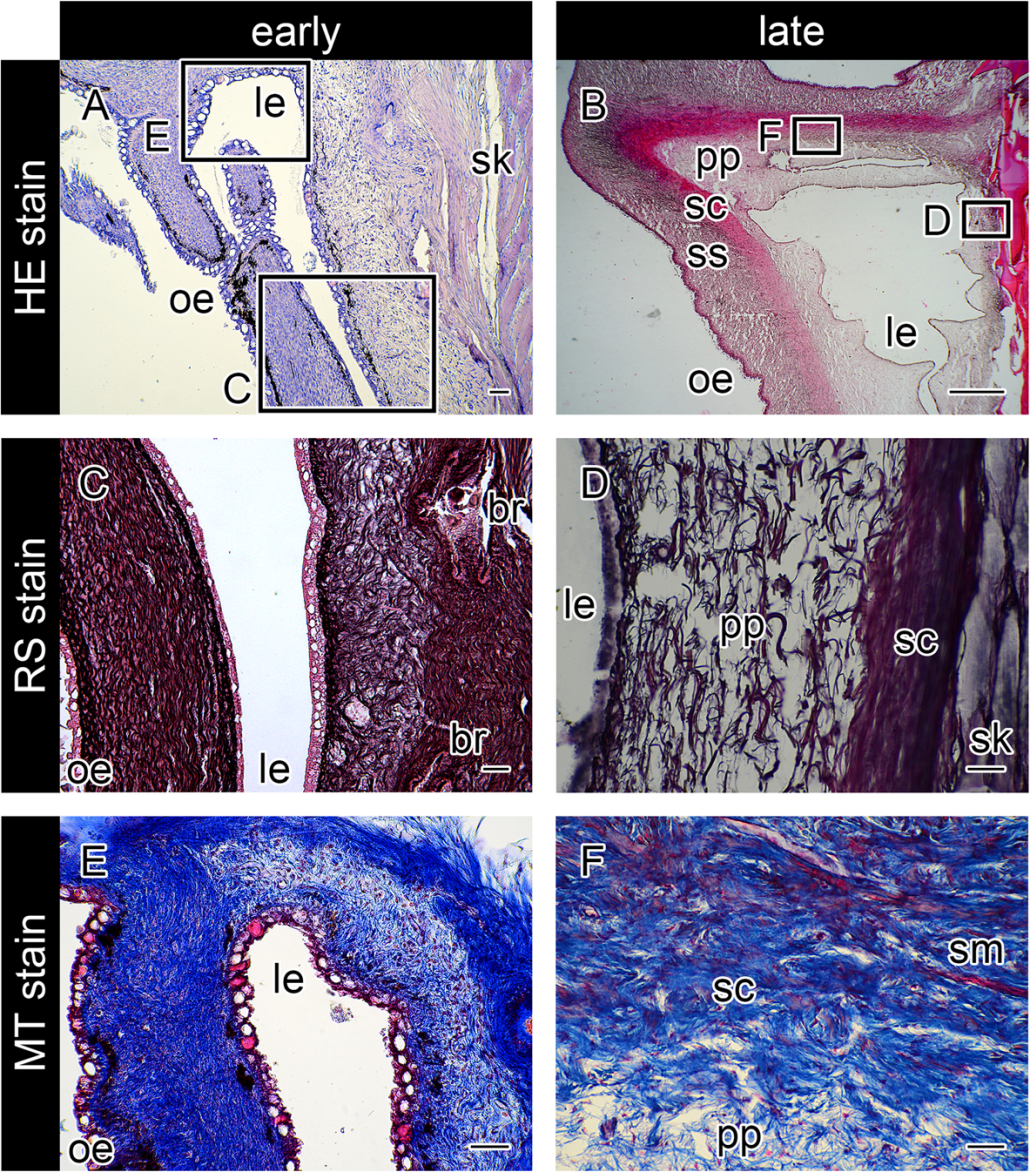
Sagittal sections of the seahorse brood pouch. Sections of the early stage just after a baggy structure is formed (**a**, **c**, **e**), and in the late stage (**b**, **d**, **f**), after hematoxylin and eosin (HE) staining (**a**, **b**), reticulin silver (RS) staining (**c**, **d**), and Masson’s trichrome (MT) staining (**e**, **f**). The ventral side is to the left and the dorsal side is to the right. The lettered boxes in **a** and **b** indicate the corresponding sites of high magnification views shown in **c-f**. Scale bars: **a**, **c**–**f** = 40 μm; **b** = 1 mm. br, body ring; le, luminal epithelium; oe, outer epithelium; pp, pseudoplacenta; sc, stratum compactum; sk, skeletal muscle; sm, smooth muscle; ss, stratum spongiosum

(1) Early stage (1–3 months, body length 4.5–7.0 cm): the brood pouch as a baggy structure is formed from the primordium. The process of formation was observed in sections after HE staining (Fig. 4). At the Y-shaped seam line of the pouch, the dermis in the region of the elongated projections was found to be a single layer (Fig. 4a, d). Dermal cell density was higher in the ventral side of the lumen than in the dorsal side, and the pseudoplacenta was not yet evident (Fig. 5a–c, Fig. 6, and Additional file 2: Figure S2). For further confirmation, reticulin silver staining and MT staining were performed. No black-colored stain was found after reticulin silver staining (Fig. 4g, Fig. 6c, and Additional file 2: Figure S2h-j), but the dermis was stained blue by MT (Fig. 4j, Fig. 6e, and Additional file 2: Figure S2 k, l). These findings suggest that the early stage brood pouch mainly comprises a single layer of dermis, while the pseudoplacenta is not yet formed. No smooth muscle is found within the dermis layer at this stage. Additionally, both the outer and luminal epithelia contained many mucous granules. These results suggest that, in the early stage of brood pouch formation, the morphological characteristics of the luminal epithelium are similar to that of the outer epithelium. These characteristics were still evident in one individual two months after birth (data not shown). In summary, development of the early-stage brood pouch can be described as follows: Formation of the brood pouch begins from the primordium, which first appears as linear projections on both ventrolateral sides posterior to the anus; the projections subsequently fuse, anteriorly to posteriorly, along the body midline. Consequently, a baggy structure forms posterior to the anus with a pore-like opening. In this early stage, the dermis of the ventral part of the pouch is constructed as a single layer, and the pseudoplacenta has not yet formed.

(2) Middle stage (3–7 months, body length 6.7–9.8 cm): various tissues began to differentiate and become visible in the mature brood pouch. At 3–5 months after birth, the dermis of the ventral side began to develop into two layers: the stratum compactum and stratum spongiosum (Fig. 4e). After MT staining, pale red coloring was evident between the stratum compactum and stratum spongiosum (Fig. 4k), suggesting the beginning of smooth-muscle formation. After reticulin silver staining, black staining could be discerned on the anterodorsal side of the pouch, suggesting the formation of the pseudoplacenta (Fig. 4h). Serial sections of the body revealed pseudoplacenta only on the anterodorsal side and not on the ventral or posterodorsal sides of the pouch (Fig. 5d, e). As development proceeds, the pseudoplacenta grows and extends toward the posterioventral side of the pouch, and during the later period of this stage it finally surrounds the entire lumen (Fig. 4c and Fig. 5g, h). Not only the pseudoplacenta, but also two dermis layers and smooth muscle became more visible in MT staining in individuals 6–7 months after birth (three individuals examined: one at six months, and two at seven months) (Fig. 4l).

(3) Late stage (6 months-, body length 9.3 cm-): the pouch fold, the structure that will hold the eggs during incubation, was formed in the pseudoplacenta (Fig. 5j). Many blood vessels were observed in the pseudoplacenta (Fig. 5k), which reportedly become especially abundant during incubation [10]. Indeed, male seahorses at 7–8 months after birth were sexually mature and could incubate embryos within their brood pouch. The well-developed smooth muscle was established mainly around the pouch entrance (Fig. 6b, and Additional file 3: Figure S3). The large mucous granules otherwise observed in the luminal epithelium at the early stage gradually decreased in number during development of the brood pouch (Fig. 5).

### Cloning of C-type lectin cDNAs

**Fig. 7:**
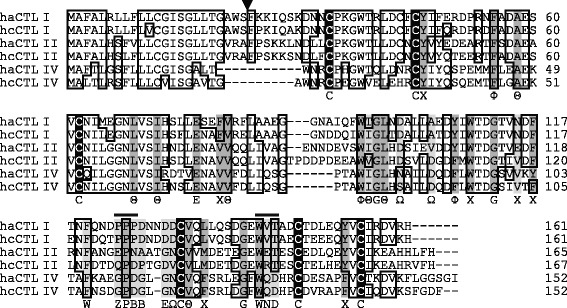
Amino-acid sequence alignment of C-type lectins (CTLs). Amino-acid sequence alignment was made from CTLs of *Hippocampus abdominalis* (i.e., haCTL I, II, and IV) together with those of *H. comes* (i.e., hcCTL I, II, and IV). Identical residues are boxed; dashes represent gaps; the triangle indicates the putative cleavage site of a signal sequence. Bars above sequences indicate the part of the Ca^2+^ binding site involved in sugar specificity. Conserved cysteine residues are shaded in black, and other conserved residues are shaded in dark gray; residues of the consensus motif that are not conserved in seahorse CTLs are shaded in light gray. Consensus residues of the lectins are shown below the sequences. Θ, aliphatic; Φ, aromatic; Χ, aliphatic or aromatic; Ω, side chain with carbonyl oxygen atom (D, N, E or Q); Z, E or Q; B, D or N [23]

Previous research reported that three kinds of group-VII C-type lectins (CTLs) (namely, hcCTL I, hcCTL II, and hcCTL III) were expressed in the brood pouch of *Hippocampus comes* [16]. Using primers designed from the conserved regions of hcCTLs, three kinds of CTL cDNAs were cloned from *H. abdominalis* by RT-PCR. Cross-species comparisons enabled us to identify the *H. abdominalis* genes homologous to *H. comes* CTL reported previously (Additional file 4: Table S1). One of these genes showed the highest similarity to hcCTL I (83%), which we therefore named haCTL I. Another gene had the highest similarity to hcCTL II (73%), and was named haCTL II. The rest of the genes studied did not show high similarity to any of the hcCTL I-III genes, and were therefore named haCTL IV. BLAST search analysis revealed that haCTL IV was similar to an unnamed gene of *H. comes* (accession number: XP_019719077) (76%), and was named hcCTL IV. The sequence analysis revealed that all the cloned haCTLs consisted of a signal sequence and 133–142 amino-acid residues of the CRD (Fig. 7). Within the CRD, six cysteine residues conserved in C-type lectins were found in all the cloned haCTLs. Two motifs (that is, EPN/QPD and WND motifs) are known to be the binding site for sugar; of these, the EPN and QPD motifs are of galactose and mannose specificities, respectively [22]. Although the three haCTLs showed an overall similarity to C-type lectins [23], only the EPN motif (not the WND motif) was found in haCTL II, and clear consensus sequences having sugar-binding motifs were not found in the regions equivalent to those of haCTL I and haCTL IV. Sequence similarity between the three haCTLs was 41–52% (Additional file 4:Table S1).

Phylogenetic analysis of fish group-VII CTLs revealed that haCTL I, II and IV and hcCTL I–IV belonged to the same clade (Additional file 1: Figure S1). In that clade, haCTL I and hcCTL I formed sister clade, haCTL II formed a subclade together with hcCTL II and other CTLs, and haCTL IV and hcCTL IV formed a sister clade. These results confirmed that haCTL I, II and IV are homologous to hcCTL I, II and IV, respectively.

### Expression of C-type lectin genes

**Fig. 8:**
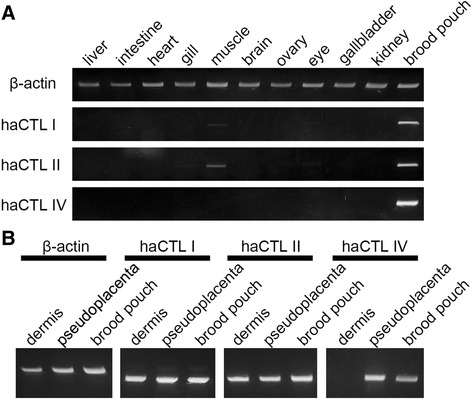
Semi-quantitative analysis of C-type lectin gene expression. **a** Semi-quantitative expression analysis of C-type lectin genes in adult seahorse liver, intestine, heart, gill, muscle, brain, ovary, eye, gallbladder, kidney, and late-stage brood pouch. **b** Comparison of C-type lectin gene expression in late-stage brood pouch tissues: dermis along with outer epithelium, pseudoplacenta along with luminal epithelium, and whole brood pouch. β-actin was used as a control

We analyzed the expression pattern of three haCTLs in various tissues or organs of three adult seahorses, using RT-PCR. As shown in Fig. 8a, all the haCTL genes were strongly expressed in late stage brood pouch tissue of a single mature male; however, the haCTL I and haCTL II genes were weakly expressed in muscle, probably due to contamination of the outer-epithelium sample during the isolation procedure of muscle RNAs (see next section). Using RNAs extracted from two regions of the brood pouch of a late stage seahorse male (dermis along with outer epithelium, and pseudoplacenta along with luminal epithelium), we examined the expression of the haCTL genes (Fig. 8b) and found that the haCTL I and haCTL II genes were expressed in both parts. In contrast, the haCTL IV gene was expressed only in pseudoplacenta along with luminal epithelium.

### Localization of the C-type lectins

**Fig. 9:**
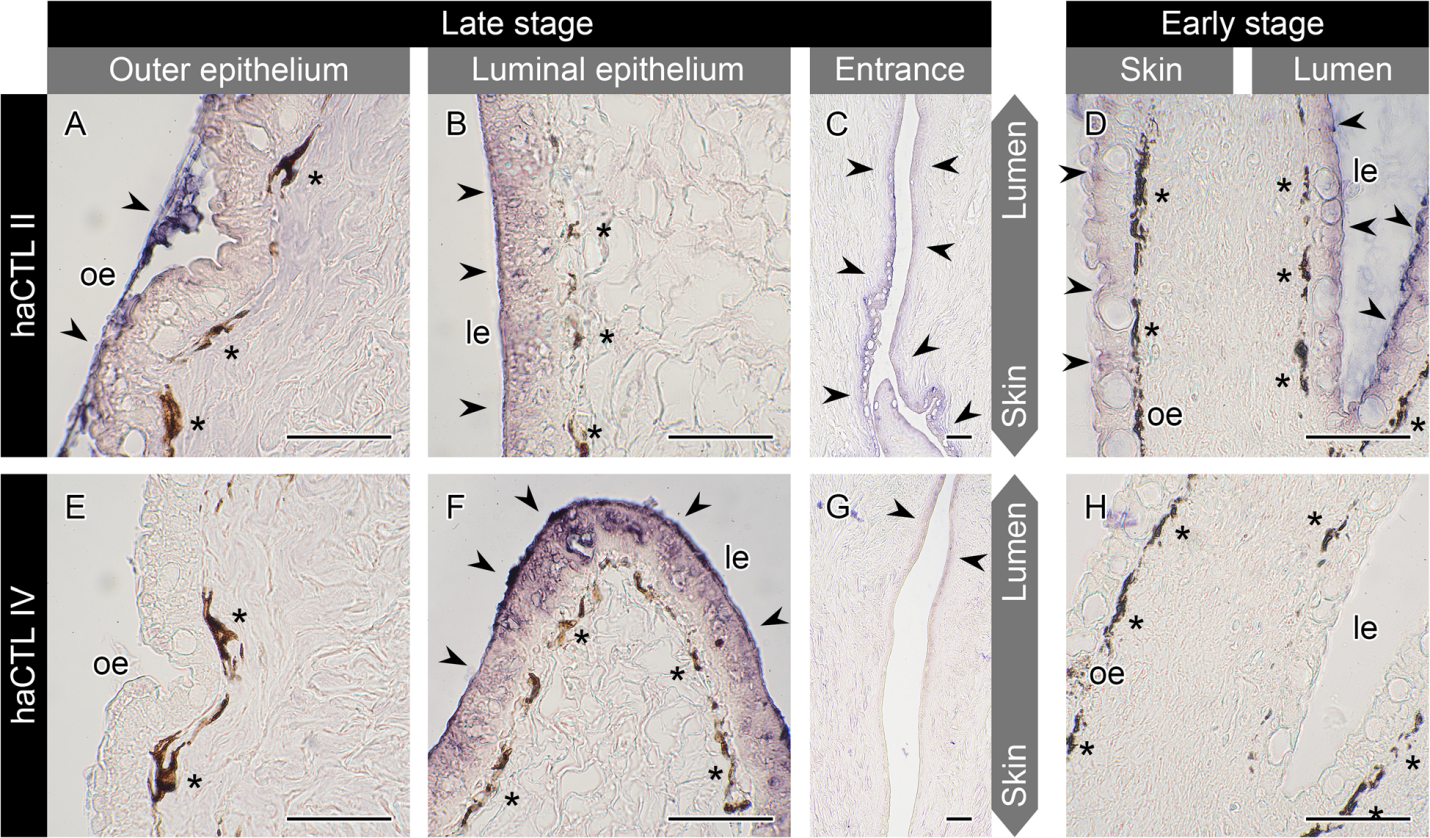
Immunohistochemical analysis of seahorse brood pouch with anti-haCTL II and anti-haCTL IV antibodies. Sections of late-stage brood pouch were immunostained with anti-haCTL II (**a**–**c**) and anti-haCTL IV (**e**–**g**) antibodies: outer epithelium (**a**, **e**), luminal epithelium (**b**, **f**), and pouch entrance (**c**, **g**). Sections of early-stage brood pouch were immunostained with anti-haCTL II (**d**) and anti-haCTL IV (**h**) antibodies. Arrowheads indicate positive signals, and asterisks indicate pigment cells. le, luminal epithelium; oe, outer epithelium. Scale bars = 50 μm. Sections for **a**–**c** and **e**–**g** were derived from one individual, and those of **d** and **h** were derived from another individual

To identify the localization of haCTL proteins, we first synthesized recombinant proteins of haCTL II and haCTL IV in *E. coli*, and hence obtained their antibodies using the recombinant proteins as antigens. Immunohistochemical staining of mature brood pouch samples from two late stage seahorses revealed that haCTL II was localized in both the outer and luminal epithelia (Fig. 9a, b), while haCTL IV was localized specifically in the luminal epithelium (Fig. 9e, f). These results are consistent with the findings of the gene expression analysis indicating that haCTL II and IV show different domains of expression (Fig. 8b).

We further examined the localization of haCTL II and haCTL IV in the region of the pouch entrance. As shown in Fig. 9c, haCTL II-positive signals were detected in the luminal and outer epithelia. Yet, the haCTL IV signals observed in luminal epithelium became weak near the middle of the entrance and then disappeared near the outer epithelium (Fig. 9g).

Lastly, we observed the localization of haCTL II and haCTL IV in the brood pouch of an early stage individual: haCTL II-positive signals were detected in both the luminal and outer epithelia (Fig. 9d), but haCTL IV-positive signals were not detected in either type of epithelia (Fig. 9h). These results suggest that haCTL IV synthesis starts in the luminal epithelium during brood pouch formation. Therefore, haCTL II shows potential for use as a marker of both luminal and outer epithelia, while haCTL IV may prove to be a useful marker of the luminal epithelium.

## Discussion

The brood pouch of male pot-bellied seahorse, *Hippocampus abdominalis*, was distinguished as comprising three layers, specifically the pseudoplacenta and two dermis layers, using three staining experiments. The pseudoplacenta was colored black after reticulin silver staining, suggesting that the pseudoplacenta is composed primarily of reticular fibers, a feature that would be appropriate for a structure supporting fragile embryos. The two dermis layers were colored blue using Masson’s trichrome staining, and a part of the outer dermis (stratum spongiosum) was colored black by Elastica van Gieson stain in two of four individuals. These results indicate that the two layers of dermis are composed mainly of collagenous fibers, and that the outer dermis contains a small amount of elastin fibers. These fiber structures lend aid to not only strength but also elasticity of the brood pouch.

During brood pouch formation, smooth muscle differentiates within the dermis, and the muscle especially abundant around pouch entrance is closely related to mating behavior. The unique courtship and mating rituals of seahorses have been described elsewhere [24]. Courtship is initiated by dilating the entrance of the brood pouch to draw in water, rendering the pouch enlarged and bloated. The male swims with a pumping action, and then closes the pouch entrance. After accepting a courting male, the female transfers eggs to the entrance of the male’s pouch. Later, at the time of delivery, the male dilates the pouch entrance and repeatedly bends his body to expel the offspring. We determined that smooth muscle is concentrated around the entrance of the pouch. The well-developed smooth muscle probably enables the pouch entrance to be opened and closed during courtship, and re-opened at the start of birthing behavior.

In this study, three types of C-type lectins (haCTL I, II and IV) were cloned from *H. abdominalis* and found to be localized in the outer epithelium and/or luminal epithelium of the male’s brood pouch. In several fishes, lectins are reportedly localized in the mucous cells of the dermis. Examples include C-type lectin AJL from eel *Anguilla japonica*, and pufflectin from pufferfish *Takifugu rubripes* [25–27]. Among these, the haCTLs showed 30% sequence identity to AJL (accession number: AB050703), but no similarity to pufflectin. AJL is suggested to be a mannose-binding lectin due to the presence of EPN and WND motifs [28]. However, the haCTLs showed sequence variation in the region corresponding to the two sugar-binding sites (Fig. 7), which are known to determine sugar specificity. These results suggest that haCTLs and AJL possess different sugar specificity, and accordingly, all the cloned haCTLs showed different sugar specificity. It has been reported that more than 15% of the 168 cloned sequences in brood pouch cDNA libraries are constructed from seahorse brood pouch-encoding lectins, including hcCTL I, II and III [16]. In particular, hcCTL III is secreted into the brood pouch during early pregnancy, and has been suggested to function in embryo-defense and/or self-defense by agglutination of microorganisms and suppression of bacterial growth [16].

The luminal epithelium is transformed drastically in gene expression and in morphology during development. Large mucous granules were observed as equally abundant in the luminal and outer epithelia in the early stage of brood pouch formation. As development proceeded, the number of mucous granules in the luminal epithelium decreased. Along with this morphological change, haCTL IV was specifically localized in the luminal epithelium of the pouch. It is assumed that the brood pouch of seahorses exerts specific functions, particularly waste removal, gas exchange, osmoregulation and protection of embryos during incubation [29]. In the mature brood pouch, accompanied with the pseudoplacenta formation, the luminal epithelium also changed to be adapted to such functions. In future studies, we intend to use molecular markers to observe development of the specific brood pouch tissues.

## Conclusions

**Fig. 10:**
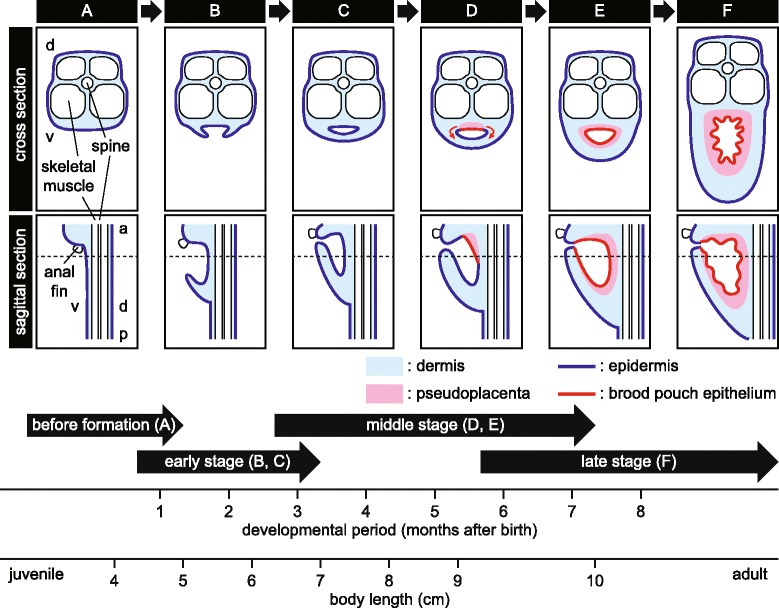
A proposed pathway of brood pouch formation in the seahorse. The pathway is schematically shown in both cross-sectional and sagittal views. **a** Before formation of the brood pouch. **b** The primordium of the brood pouch first appears as linear projections at both ventrolateral sides of the body. **c** The projections become fused at the body midline. **d** Differentiation of brood pouch tissue begins, and the pseudoplacenta first appears on the dorsal side of the pouch. **e** The pseudoplacenta eventually surrounds the pouch entirely. **f** Finally, the pouch-folds are formed, ready to incubate embryos. Dotted lines in sagittal views indicate locations of cross-sectional views. The typical developmental periods of the brood pouch stages and body length are given at the bottom of the figure. Formation may differ between individuals depending on rearing temperature and/or nutrition. a, anterior; d, dorsal; p, posterior; v, ventral

The present study provides basic information on the morphology of brood pouch formation during seahorse development and establishes a foundation for further study of the molecular mechanisms of brood pouch formation. Based on our observations of the various stages of brood pouch formation in *Hippocampus abdominalis* (Figs. 3, 4, 5 and 6, and Additional file 2: Figure S2 and Additional file 3: Figure S3), we propose to classify the process of brood pouch formation into early, middle and late stages (Fig. 10). The early stage (1–3 months) entails the formation of a primary baggy structure, wherein the primordium of the brood pouch first appears as raised linear projections on both ventrolateral sides of the body. The projections become fused at the body midline. Finally, a small pit left posterior to the anus as a pore-like pouch entrance. Moreover, in this early stage, the pouch is surrounded only by dermis, and neither smooth muscle nor pseudoplacenta are formed. The middle stage (3–7 months) involves differentiation of brood pouch-specific tissues. The pseudoplacenta first appears on the dorsal side of the pouch, and eventually surrounds the pouch entirely, and the dermis acquires smooth muscle and is composed of two layers. The late stage (from six months) is that of the fully developed brood pouch, wherein the pouch-fold is complete and ready to incubate embryos.

## Additional files

Additional file 1: **Figure S1.** Maximum-likelihood tree of C-type lectins. *Hippocampus abdominalis* genes are shown in bold characters. Numbers at the nodes indicate bootstrap values shown as percentages. GenBank accession numbers of sequences used in the present study are shown right to the species name. (EPS 1677 kb)

Additional file 2: **Figure S2.** Sagittal sections of the brood pouch at the early stage. Serial sagittal sections of the body at an early stage of brood pouch formation, after hematoxylin and eosin staining (**a**-**g**), reticulin silver staining (**h**-**j**), and Masson’s trichrome staining (**k**, **l**). The ventral side of the body is on the left and the dorsal side on the right. The lettered boxes in **a**, **h**, and **k** indicate sites of the high-magnification views. Scale bars: **a**–**f**, **h**, **k** = 100 μm; **g**, **i**, **j**, **l** = 20 μm. (TIFF 12606 kb)

Additional file 3: **Figure S3.** Sagittal sections of the brood pouch at the late stage. Serial sagittal sections of the body at the late stage of brood pouch formation were observed after hematoxylin and eosin staining (**a**–**f**) together with their high magnification view of pseudoplacenta (**i**) and dermis (**j**). Sections were further observed using reticulin silver staining (**g**, **k**), and Masson’s trichrome staining (**h**, **l**). The ventral side of the body is on the left and the dorsal side on the right. The lettered boxes in **d**, **e**, **g**, and **h** indicate sites of high magnification. Scale bars: **a**–**h** = 1 mm; **i**–**l** = 40 μm. br, body ring; pp, pseudoplacenta; sc, stratum compactum; sm, smooth muscle; ss, stratum spongiosum. (TIFF 39781 kb)

Additional file 4: **Table S1.** Amino acid sequence similarity between *Hippocampus abdominalis* (haCTL I, II, and IV) and *H. comes* (hcCTL I-IV) C-type lectins. (DOCX 16 kb)
