# Supplementary figures and images for: Morphology of brood pouch formation in the pot-bellied seahorse Hippocampus abdominalis
Source: Zoological Lett. 2017 Oct 17;3:19. doi: 10.1186/s40851-017-0080-9 (PMC5646163; doi:10.1186/s40851-017-0080-9)

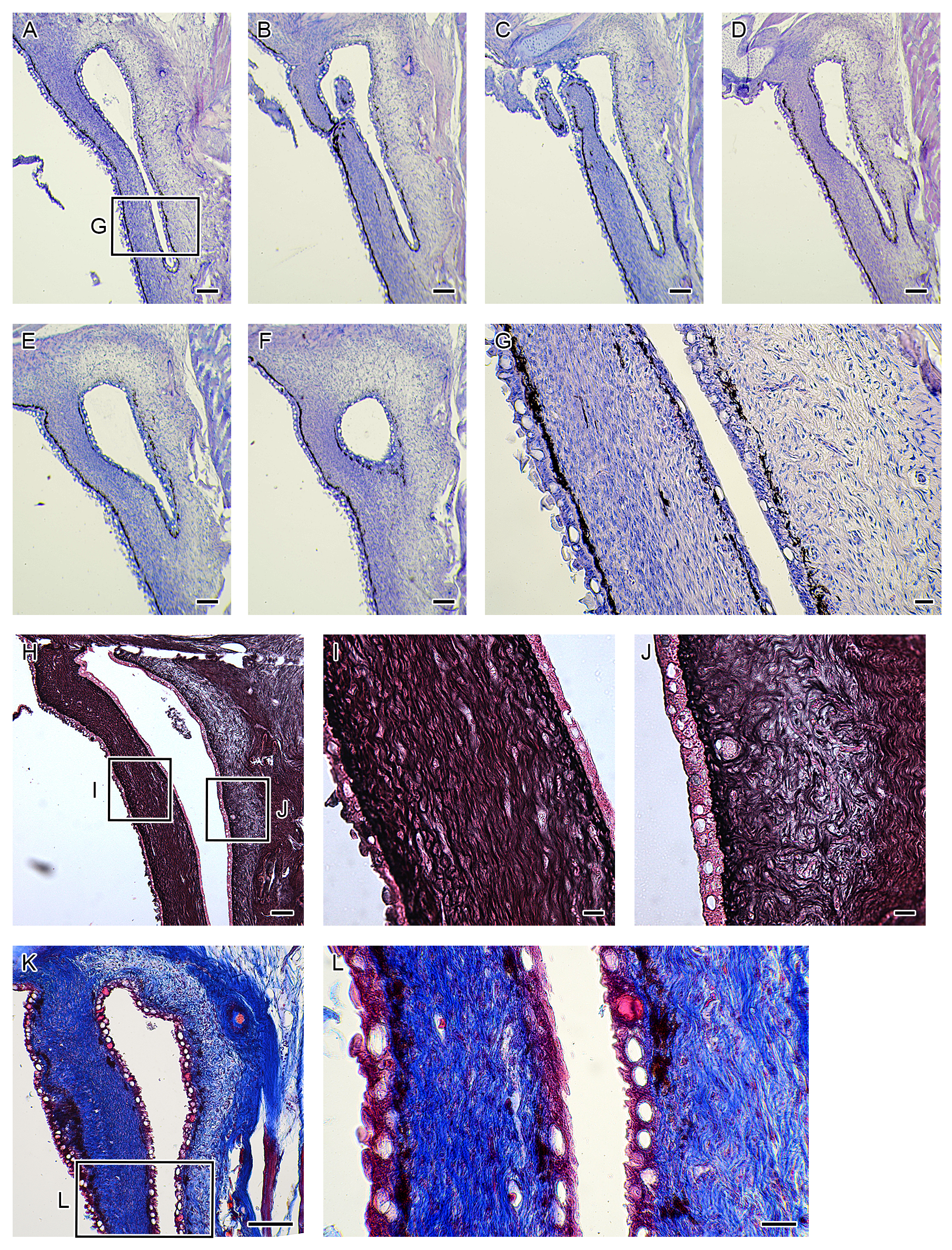

Supplement: Supplementary file 2 — Figure S2. Sagittal sections of the brood pouch at the early stage. Serial sagittal sections of the body at an early stage of brood pouch formation, after hematoxylin and eosin staining (a-g), reticulin silver staining (h-j), and Masson’s trichrome staining (k, l). The ventral side of the body is on the left and the dorsal side on the right. The lettered boxes in a, h, and k indicate sites of the high-magnification views. Scale bars: a–f, h, k = 100 μm; g, i, j, l = 20 μm. (TIFF 12606 kb) [file 40851_2017_80_MOESM2_ESM.tif]

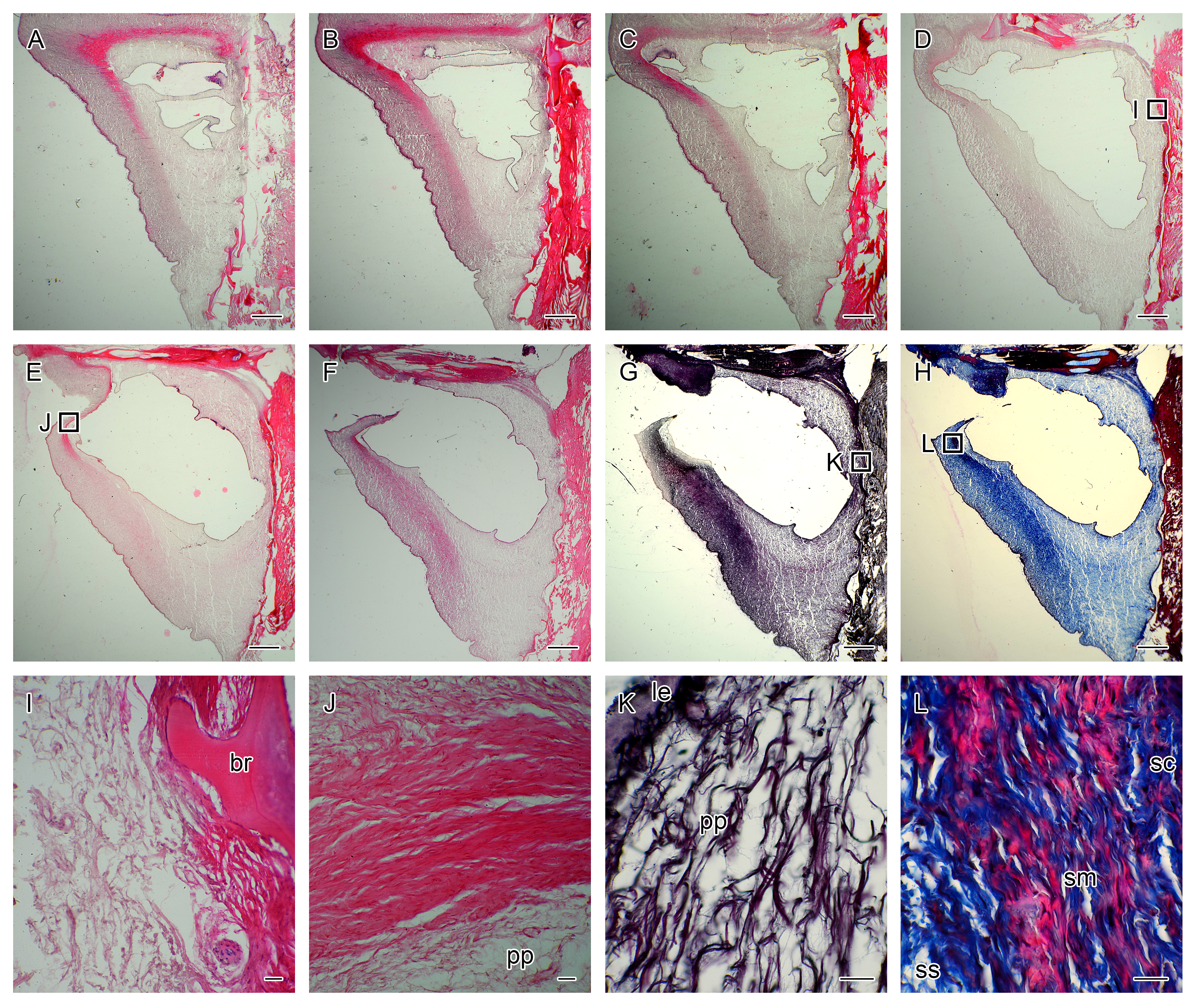

Supplement: Supplementary file 3 — Figure S3. Sagittal sections of the brood pouch at the late stage. Serial sagittal sections of the body at the late stage of brood pouch formation were observed after hematoxylin and eosin staining (a–f) together with their high magnification view of pseudoplacenta (i) and dermis (j). Sections were further observed using reticulin silver staining (g, k), and Masson’s trichrome staining (h, l). The ventral side of the body is on the left and the dorsal side on the right. The lettered boxes in d, e, g, and h indicate sites of high magnification. Scale bars: a–h = 1 mm; i–l = 40 μm. br, body ring; pp, pseudoplacenta; sc, stratum compactum; sm, smooth muscle; ss, stratum spongiosum. (TIFF 39781 kb) [file 40851_2017_80_MOESM3_ESM.tif]
