# Supplementary material for: Morphology of brood pouch formation in the pot-bellied seahorse Hippocampus abdominalis
Source: Zoological Lett. 2017 Oct 17;3:19. doi: 10.1186/s40851-017-0080-9 (PMC5646163; doi:10.1186/s40851-017-0080-9)
Supplement: Supplementary file 4 — Table S1. Amino acid sequence similarity between Hippocampus abdominalis (haCTL I, II, and IV) and H. comes (hcCTL I-IV) C-type lectins. (DOCX 16 kb) [file 40851_2017_80_MOESM4_ESM.docx]

Supplementary Table 1. Amino acid sequence similarity between *H. abdominalis* (haCTL I, II, and IV) and *H. comes* (hcCTL I-IV) C-type lectins.

|  | haCTL I | haCTL II | haCTL IV |
| --- | --- | --- | --- |
| haCTL I | - | 52 | 41 |
| haCTL II | 52 | - | 44 |
| haCTL IV | 41 | 44 | - |
| hcCTL I | **83** | 53 | 43 |
| hcCTL II | 53 | **73** | 45 |
| hcCTL III | 77 | 51 | 42 |
| hcCTL IV | 43 | 44 | **76** |
